# Association between social distancing and incident microvascular events among individuals with diabetes mellitus: a population-based cohort study

**DOI:** 10.1192/j.eurpsy.2024.645

**Published:** 2024-08-27

**Authors:** Y. Y. Liang, Y. He

**Affiliations:** ^1^Center for Sleep and Circadian Medicine, The Affiliated Brain Hospital of Guangzhou Medical University, Guangzhou, China

## Abstract

**Introduction:**

Social isolation and loneliness have been rising social determinants of cardiometabolic health.

**Objectives:**

To investigate the associations of social isolation and loneliness with diabetic microvascular complications (DMC) among individuals with type 2 diabetes mellitus (T2DM) and assess the extent to which intermediate risk factors explained the associations.

**Methods:**

Data for individuals with T2DM (n=24,297, 62.2% male; mean age=60.0 years) were taken from the UK Biobank. Social isolation and loneliness were assessed using self-reported questionnaires. DMC, mainly including diabetic kidney disease, diabetic retinopathy, and diabetic neuropathy, were identified by linking hospital records and death registries.

**Results:**

In the multivariate-adjusted model, social isolation was associated with an increased risk for incidence of any DMC (most vs. least: HR: 1.13; 95% CI: 1.05-1.22), especially diabetic kidney disease and neuropathy; loneliness was also associated with any DMC (yes vs. no: HR: 1.12; 95% CI: 1.02-1.23) and diabetic kidney disease. Social isolation and loneliness ranked similarly in relative strength for predicting DMC as other conventional risk factors, such as smoking, high blood pressure, and physical activity. The association between social isolation and DMC was mainly attributed to health behaviors, while the association between loneliness and DMC was primarily explained by health behaviors, psychological factors, and diabetes-related factors.

**Conclusions:**

Social isolation and loneliness were independently associated with a higher risk for incident DMC among individuals with T2DM, which were largely explained by subsequent unhealthy lifestyles, psychosocial stress, and diabetes-related factors. These findings underscore social isolation and loneliness as novel modifiable risk factors for predicting DMC.

**Acknowledgements:**

This research has been conducted using the UK Biobank Resource under Application Number 58082.

**Funding Support:**

This work was supported by the National Natural Science Foundation of China (grant number 32100880), Guangzhou Municipal Key Discipline in Medicine (2021-2023), Guangzhou High-level Clinical Key Specialty, and Guangzhou Research-oriented Hospital. The funders had no role in the design and conduct of the study; collection, management, analysis, and interpretation of the data; preparation, review, or approval of the manuscript; and decision to submit the manuscript for publication.

**Disclosure of Interest:**

None Declared

